# Community pharmacists’ views on the implementation of a national electronic prescription transfer system to pharmacies: A cross-sectional survey

**DOI:** 10.1016/j.rcsop.2026.100826

**Published:** 2026-07-29

**Authors:** Kieran Dalton, Ciara Kenny, Maeve Culhane, Robert Callaghan

**Affiliations:** Pharmaceutical Care Research Group, School of Pharmacy, University College Cork, Cork, Ireland

**Keywords:** Community pharmacy, Prescription, Communication, Pharmacist, General practitioner, Primary care, Survey

## Abstract

**Introduction:**

Legislation was implemented in Ireland in April 2020 to permit electronic prescription transfer (EPT) from prescribers directly to pharmacies using a national secure email system (Healthmail). With a clear need to assess how this initiative affected patient care delivery, this study aimed to evaluate community pharmacists' views of EPT to pharmacies via Healthmail.

**Methods:**

A cross-sectional online survey was disseminated via email in November 2020 to all Pharmaceutical Society of Ireland pharmacist registrants with ‘community pharmacy’ as their practice area (*n* = 3780). Descriptive statistics were performed on closed-ended questions. Free-text comments underwent conventional content analysis.

**Results:**

From 494 responses, most agreed it was an efficient and convenient method of prescription transfer (89.7%), 81.8% agreed it had successfully integrated into dispensary workflow, and 75.8% preferred receiving prescriptions in this way compared to other methods. While 94.7% agreed it was a positive step for pharmacy practice, key points from the open comments included concerns with identifying the sender of prescriptions and the need to better manage patient expectations on prescription processing times. The majority believed that patients did not have enough information about Healthmail (70.5%) and 63.2% perceived that it has led to patients feeling less involved in the decision-making related to their care.

**Conclusion:**

This study indicates that pharmacists were mostly positive about the national introduction of EPT to pharmacies, while also perceiving concerns regarding its potential negative impact on patients' medication management. Further guidance for all stakeholders should be considered a high priority when implementing EPT to achieve best practices.

## Highlights

• Pharmacists welcomed the introduction of electronic prescription transfer.
• Insufficient stakeholder education caused unrealistic expectations for dispensing.
• With direct pharmacy transfer, patients were less aware of prescription content.
• Pharmacists believed some prescriptions were sent without prescriber review.
• Future preference for retrieving prescriptions from shared online health record.

## 1 Introduction

Medication prescribing has historically been based on physical prescriptions that patients had to take from prescribers to pharmacies. This use of physical prescriptions dates back over 4000 years, with seemingly the earliest known prescriptions documented on clay tablets in ancient Mesopotamia.^1^ Physical prescriptions with handwriting have long been used but have a number of shortcomings, including, but not limited to, the potential for dispensing errors due to illegibility and susceptibility to forgery.^2^ Although the introduction of printing and computers enabled the generation of typed prescriptions which reduced errors related to handwriting, patients typically still had the burden of transporting the prescription to a pharmacy and waiting for it to be dispensed.^3^ However, over the last two decades, there has been a push for prescriptions to be transferred electronically – helping to overcome some of the problems with physical prescriptions.^4^ Countries such as the United Kingdom, United States of America, Finland, and Denmark have pioneered the implementation of such initiatives with great success.^5^ Physicians and pharmacists have expressed satisfaction with the implemented electronic systems, especially as they have decreased dosage and formulation errors, and improved patient care overall.6., 7.

In Ireland in 2014, a new national secure email system called Healthmail was implemented, which was initially used as a means of communicating patient-identifiable clinical information between physicians and hospitals (e.g. letters or results of tests or procedures). A survey was conducted in 2017 to evaluate general practitioners' (GPs') level of satisfaction with Healthmail, and opinions were resoundingly positive, which helped contribute to Healthmail's expanded use to community pharmacists, dentists, nursing homes, and optometrists.^8^ However, as electronic prescription transfer (EPT) was not permitted at the time, this system was not widely used by pharmacists, and pharmacists still required a physical copy of the prescription to be provided for dispensing. This all changed when COVID-19 was declared a global pandemic by the World Health Organization. With the need to minimise in-person interactions and ensure continued medication supply to patients, April 2020 saw the implementation of legislation in Ireland to permit EPT from a prescriber to community pharmacies using Healthmail,^9^ without the need for a signed paper prescription copy, as was previously required.

Since the introduction of these changes, there has been a substantial increase in the volume of prescriptions being sent electronically in Ireland.^10^ As an example to reflect this: 64,025 emails were sent using Healthmail in January 2020, which surged to a monthly total of 808,938 by June 2020, and increased to a monthly total of 2,105,000 by June 2025 – of which 1,807,835 were sent to pharmacies, demonstrating a big shift in healthcare communication pathways.

When significant changes occur to prescription use, it is vital to assess the impact on all key stakeholders, with a particular focus on how it affects patient care. The main stakeholders affected by the prescription and its journey are the prescriber, the patient, and the pharmacist – often referred to as the three 'P's of prescribing.11., 12. As mentioned previously, prescriber satisfaction with Healthmail was high, with studies amongst physicians in other countries where EPT has been implemented showing similar results.8., 13., 14. Similarly, patients have expressed satisfaction with EPT in multiple countries, touting reduced wait times, increased satisfaction, and superior care quality.15., 16., 17. Pharmacists are undoubtedly a vital stakeholder group, especially as approximately 86% of total Healthmail emails sent in June 2025 were sent to pharmacies (1,807,835 of 2,105,000), and pharmacists are the last stakeholder to review prescriptions before the patient receives their medication.18., 19., 20. Given the need to gather key stakeholders’ views, this study aimed to evaluate Irish pharmacists' perspectives on the introduction of EPT via Healthmail, its impact on community pharmacy practice and patient care, as well as the implications for the future use of EPT.

## 2 Methods

Study reporting was guided by the Checklist for Reporting of Survey Studies (CROSS) and Strengthening the Reporting of Observational Studies in Epidemiology (STROBE) Statement.21., 22.

### 2.1 Study context for healthmail and EPT to Irish community pharmacies

Healthmail is funded by Ireland's national provider of health and social care services, the Health Service Executive (HSE). Healthmail servers are securely connected, using Transport Later Security (TLS), and emails can only be sent to and received by specifically approved accounts within the server. Generally, each pharmacy will have one Healthmail account, whereas general practices may be more likely to have more than one account associated with the practice (e.g. one for the practice itself, and individual GPs may have their own accounts). Healthmail accounts may be accessible to non-pharmacist staff in pharmacies (e.g. pharmacy technicians) and non-GP staff in GPs' practices (e.g. secretary). While most prescriptions via Healthmail are sent from prescribers to pharmacies, it is possible for pharmacies to forward a prescription to another pharmacy (e.g. in the event a patient wishes to change pharmacy or a prescriber accidently sends the prescription to the wrong pharmacy). However, Ireland's pharmacy regulator outlines that “*prescriptions can only be forwarded to another pharmacy in very exceptional circumstances to facilitate continuity of patient care*”.^23^

Healthmail was integrated within GPs' prescribing software in 2020, which allowed for a seamless transfer of prescriptions that have just been generated in the software. Upon the allowance of EPT to pharmacies, joint guidance was produced by the HSE and professional regulatory bodies for physicians and pharmacists – the Medical Council and Pharmaceutical Society of Ireland (PSI) respectively.^9^ However, no formal nationwide training was provided regarding Healthmail usage.

Prescriptions provided via Healthmail could be sent as part of the body of the email (e.g. as written text) or as an attachment (e.g. PDF). These prescriptions still had to meet the same legal requirements as a physical prescription, with the exception of a handwritten signature, and had to be electronically traceable back to the prescriber. At the time of the survey, pharmacists had to print a copy of each electronic prescription and treat it as the original prescription for record-keeping purposes.

Beyond the transfer of prescriptions, pharmacists could send emails to prescribers when they have queries about prescriptions or when making an intervention regarding a prescription, for example by replying to the thread containing the patient's prescription.

### 2.2 Survey design

The cross-sectional survey content was constructed by researchers CK and KD, informed by a comprehensive review of relevant literature8., 24., 25., 26., 27., 28. and the researchers' experience of working in the community pharmacy setting. At the time of conducting this study, KD was a pharmacist based in academia with six years of post-qualification experience, and CK was a final year pharmacy student. To minimise either researcher bringing significant personal biases, informal discussions around Healthmail also took place with practising community pharmacists to inform the survey content, with reflexivity being encouraged throughout the study. The survey was then piloted with two practising community pharmacists, who reviewed the survey for face and content validity.

The final survey (**Appendix A**) consisted of 33 questions, divided into four sections: i) respondent demographics, ii) the pharmacist's experience of using Healthmail, iii) impact of Healthmail on the patient, and iv) implications for future practice. Question types included multiple choice style questions, yes/no questions, ranking questions, Likert scale ratings, and open comment sections. An open comment box was provided to allow participants to provide their gender, ‘other’ reasons for choosing not to conduct an intervention with the prescriber via Healthmail, and for each of the survey's final two questions: i) asking how could the process of EPT be improved, with a request for up to three suggestions, and ii) asking to include any other thoughts or comments regarding issues addressed in this survey.

### 2.3 Survey distribution

Ethics approval for the study was granted by the School of Pharmacy Social Research Ethics Committee, University College Cork. Pharmacists who had practised full time or part time in a community pharmacy setting, in Ireland, since the implementation of Healthmail, were invited to participate in the study via email. An email containing the link to the survey was sent to all pharmacists listed on the PSI's email list who had listed ‘community pharmacy’ as their area of practice (*n* = 3780). This email was sent on 10th November 2020. On the same date as the survey release, a notice was posted on the Pharmabuddy® website to encourage pharmacists to check their email for the link to the survey. Pharmabuddy® is a pharmacist education and support network, which has a forum regularly used by pharmacists. This forum post did not contain the survey link, but provided the research team's contact details to allow potentially eligible pharmacists who had not received the email to request the survey link.

Participation in the study was voluntary. On accessing the survey link, an information sheet and consent form preceded the survey questions. Participants could withdraw from the study up until the point of data submission. A reminder email was sent to all 3780 pharmacists on 24th November 2020. Based on the research team's experience with conducting online surveys with pharmacists in Ireland, the response rates typically decrease substantially after reminder emails. The survey was closed to responses on 8th December 2020 as only seven responses had been received in the preceding seven days; leaving the survey open beyond this time period was not considered necessary.

### 2.4 Data analysis

The data were extracted from the survey platform (Microsoft® Forms) and then reviewed so any identifiable information could be removed.

Respondent demographics were analysed using IBM® SPSS version 26. Descriptive statistics were used to analyse demographic characteristics and responses to the various statements and questions, with the percentage frequency calculated based on valid responses (i.e. excluding missing data). Chi square analysis was conducted to assess for significant differences between the age and gender of the respondents versus pharmacists nationally at the time of the survey. Differences were considered statistically significant where *p* < 0.05.

The percentage survey response rate was calculated by dividing the number of responses received by the number of pharmacists who were sent the survey via email, and then multiplying by 100. Similarly, the percentage response rate for each of the final two questions was calculated by dividing the number of respondents who provided an answer to each question by the total number of survey respondents, and then multiplying by 100.

The responses from the two final open-ended survey questions were combined and uploaded to NVivo® version 12 to facilitate analysis. The responses were then reviewed and underwent conventional content analysis by KD and MC, which consisted of a six-step process of (i) data familiarisation, (ii) generating initial non-hierarchical codes through open coding, (iii) code categorisation to form initial themes, (iv) reviewing and discussing themes, (v) naming and defining themes, and (vi) theme write-up.^29^

## 3 Results

A total of 494 responses were received (13.1% response rate), with respondent demographics shown in Table 1.

**Table 1:** Respondent demographics.

	*%*		*%*
Gender		Pharmacy setting most frequently worked in	

Female Male	62.80%	An independent pharmacy	62.80%
Other response	36.80%	Pharmacy in a small chain (<10 pharmacies) Pharmacy in a large chain (≥10 pharmacies)	36.80%
	0.50%		0.50%

**Age**		**Pharmacy location most frequently worked in**	
<25 years	4.30%	Rural/village Town/suburb City	19.50%
26–35 years	23.40%	Shopping centre (in a town/city)	52.70%
36–45 years	32%	Shopping centre (periphery of a town/city) Other	15.00%
46–55 years	25.60%		6.90%
56–65 years	12.7		4.30%
66+ years	2%		1.60%

**Post-registration experience in community pharmacy**		**Approximate distance from the pharmacy to the nearest GP clinic**	
Less than 1 year 1–3 years		Located in the same building Within 100 m	
4–10 years	2.60%	Within 500 m Within 1 km Within 2 km	11.90%
11–20 years	4.50%	>2 km	28.10%
21–30 years	21.70%		32.60%
≥31 years	28.60%		17.40%
	29.00%		4.90%
	13.60%		5%

**Role**⁎		**Confidence using technology**	

Pharmacy owner Superintendent pharmacistΔ Supervising pharmacist† Pharmacy store manager Support pharmacist‡	29.40%	Extremely confident Very confident Moderately confident Slightly confident	31.60%
Locum pharmacist	28.50%	Not at all confident	40.50%
	48.40%		25.30%
	11.30%		1.60%
	29.10%		1%
	13.00%		

⁎ Respondents could have chosen more than one option.

Δ The superintendent pharmacist is in overall control of the management of a pharmacy, including its professional and clinical management and administrative management of the sale and supply of medicines. A superintendent pharmacist can act in respect of more than one pharmacy (i.e. all pharmacies within a company/chain) and must have at least three years' post-registration experience.

† The supervising pharmacist is the person responsible for the day-to-day management and operation of the pharmacy. A supervising pharmacist can only act in respect of one pharmacy premises and must have at least three years' post-registration experience.

‡ A support pharmacist works under the supervising pharmacist and may be responsible for the safe and effective running of the pharmacy in the supervising pharmacist's absence.

At the time of the survey, 73.8% indicated at least 7/10 prescriptions were received via Healthmail, but only 4.8% indicated at least 7/10 hospital prescriptions were sent via Healthmail.

When comparing the sample to the population of registered pharmacists in Ireland at the time of the survey (i.e. the data available, and not just community pharmacists), the population proportion of females (64.75%) and males (35.25%) was not significantly different (*p* > 0.05); however, the population age was significantly younger compared to the sample (*p* > 0.001), whereby 35.9% of the population was aged 26–35 years and 16.6% aged 46–55 years.

The overall survey question completion rate was 98.2% excluding the open-ended questions at the end of the survey.

### 3.1 Who checks Healthmail and when

When asked to rank the pharmacy staff member who most commonly checked the Healthmail account, the most frequently chosen first option was the pharmacist respondent themselves (63.9%), followed by a pharmacy technician (23.9%), and then another pharmacist (7.7%). Regarding when pharmacy staff check the Healthmail account, the percentage frequency with which respondents ranked one of four options as the first option is as follows: ‘*periodically*’ (38.6%), ‘*when the alert sounds*’ (29.7%), ‘*at set times during the day*’ (20.5%), and ‘*when a patient requests a prescription and they inform us it has been sent to the pharmacy via Healthmail*’ (11.2%).

### 3.2 Seeing the prescription before the patient

Regarding pharmacy staff seeing the prescription before the patient or patient representative presents to the pharmacy, 67.3% indicated that this occurs for at least 7/10 Healthmail prescriptions; the perceived impact of this was as follows:

• 78.9% agreed they felt more prepared to counsel the patient or their representative (6.6% disagreed).
• 66.1% agreed this improved patient safety (7.7% disagreed).
• 67.9% agreed they could inform the patient if an item is not in stock before they present to the pharmacy; 14% disagreed.
• 81.6% agreed this can improve patient satisfaction; 6.6% disagreed.
• 58.6% agreed they noticed an increase in prescription items being dispensed and not collected (22.2% disagreed).

### 3.3 Ease of use and impact on workflow and workload

Most pharmacists found Healthmail easy to use overall (92.5%), and agreed that it was an efficient and convenient method of prescription transfer (89.7%) as well as helping implement COVID-19-related infection control procedures in the pharmacy (78.2%). Even though only 44.6% agreed that the pharmacy they worked in had a designated standard operating procedure for Healthmail or EPT at the time, most felt it had been successfully integrated into dispensary workflow (81.8%). However, fewer pharmacists agreed that Healthmail had helped to streamline the dispensing process (62.4%). Although most preferred receiving prescriptions via Healthmail than other methods (75.8%), 58.5% felt Healthmail had added to their personal workload and 56.4% perceived that it added to their colleagues' workload. Only 10.3% agreed that they felt more at risk of making dispensing errors when dispensing Healthmail prescriptions (70.7% disagreed).

### 3.4 Prescription content, legibility, and comparison to physical prescriptions

While most agreed that Healthmail prescriptions were easier to read (89.5%), 22.6% often had difficulty identifying the prescriber. Over half agreed that Healthmail prescriptions often lacked one or more legal requirements (54.3%), whilst only 44.5% agreed that Healthmail prescriptions containing controlled drugs generally met all legal requirements. In contrast to this, 41.3% agreed that prescribers included extra information with Healthmail prescriptions to improve patient care (e.g. information regarding dose changes) more commonly than with physical prescriptions. Furthermore, over one third agreed that they have noticed less medication-related errors made by prescribers on prescriptions issued by Healthmail in comparison to physical prescriptions (35.6%).

### 3.5 Pharmacist-prescriber communication and pharmacist interventions

Nearly two-thirds agreed that Healthmail had improved communication between pharmacists and prescribers (64.6%), and around half agreed that Healthmail had worked well for EPT from out-of-hours’ doctor services (49.2%). Only 12.7% agreed that they received confidential patient information via Healthmail that they did not request (e.g. laboratory parameters), other than prescriptions.

Regarding the number of interventions made with the prescriber, half said this had increased in comparison to physical paper prescriptions (37.3% large increase and 13% small increase), 14.5% said there was a decrease, whilst 35.2% perceived no change.

Most pharmacists had replied to a Healthmail email to conduct an intervention (90.7%). Of these, 64.8% agreed interventions conducted via Healthmail had the same outcome as traditional methods (e.g. telephone), but only half agreed that a resolution was generally reached in a timely manner (51%); furthermore, 81.9% agreed that for some interventions they did not receive a response and had to take further action. Regarding electronic communication, 89.7% agreed that it was an appropriate way to conduct minor interventions (e.g. missing legal requirement not related to a controlled drug), 60.4% agreed it was an appropriate way to deal with clinical issues (e.g. drug-drug interaction), whilst only 20.6% agreed it was appropriate way to contact prescribers when the matter is urgent.

For the minority that did not conduct an intervention via Healthmail (9.3%), the reason for not doing so included not thinking they would get a response in a timely manner (71.7%), feeling that electronic communication was not an effective way to conduct interventions with prescribers (28.3%), and knowing that the prescribers they most commonly communicate with did not respond to pharmacist interventions via Healthmail (28.3%).

### 3.6 Prescription security

Most agreed it was a secure method of prescription transfer (91.8%) and that it reduced the number of prescriptions that have ‘*gone missing*’ or ‘*gotten lost*’ (82%). Most agreed (92.8%) that Healthmail prescriptions are less likely to be forged, and only 16.8% were worried that paperless prescriptions may facilitate others inappropriately requesting/collecting medications not meant for them (56.3% disagreed). Whilst 30% agreed that Healthmail increases the risk of people inappropriately accessing confidential patient information (e.g. other staff members in healthcare settings), 47.5% disagreed.

### 3.7 Inter-pharmacy prescription transfer

When pharmacists were posed with the scenario that another pharmacy had emailed a prescription to their pharmacy via Healthmail at the request of the patient as it had been sent to the first pharmacy in error, 65.3% indicated that they were happy to dispense the medication from the email. Of the remainder who were not happy to dispense the medication initially (34.7%):

• 53% indicated they would contact the prescriber to ask them to send the prescription to the pharmacy via Healthmail before dispensing the medication (18.4% of total).
• 42.4% indicated they would ask the patient to contact the prescriber to send the prescription to the pharmacy via Healthmail before dispensing the medication (14.7% of total).
• 4.6% indicated they would ask the patient to go to another pharmacy (1.6% of total).

### 3.8 Record-keeping and reimbursement

When asked if Healthmail had made complying with record-keeping requirements more difficult, there was a similar proportion agreeing (39.6%) to those disagreeing (41.5%). Most pharmacists agreed that pharmacies should be remunerated for printing supplies associated with using Healthmail (89.5%). Meanwhile, 44% agreed that having Healthmail prescriptions had streamlined procedures for obtaining reimbursement from the government (26.3% disagreed).

### 3.9 Training and support with Healthmail use

Only 44% agreed they received adequate information and training on the use of Healthmail. Similarly, just over half agreed that the joint HSE/PSI/Medical Council guidance for pharmacists and prescribers on the electronic transfer of prescriptions via Healthmail was easy to follow (52.6%).^9^

Very few agreed that they experienced a lot of technical issues using Healthmail in the month prior to the survey (9.3%). When asked about the quality of support provided by the Healthmail team, half required this support (50.5%); of this, 79.4% said the support was helpful/very helpful, with the remainder indicating that it was not at all helpful.

### 3.10 Impact on the patient

About two-thirds agreed that patients had adapted well to Healthmail (64.5%). Most agreed that Healthmail had facilitated a decrease in patient waiting times in the pharmacy (77.3%) and 68.6% agreed it had improved patient satisfaction. However, 80% perceived that patients expected their prescriptions to be dispensed more quickly and 70.5% agreed that patients did not have enough information about Healthmail. Furthermore, 63.2% thought patients would become less aware of the legal prescription requirements due to the ETP.

Whilst just over half (52.6%) agreed that Healthmail had a positive effect on communication between pharmacy staff and patients, 35.6% perceived there was no effect. Over half (55.4%) agreed that there had been no change in time spent on patient consultations for Healthmail prescriptions compared to consultations for physical paper prescriptions, while 34.7% perceived that there had been an increase in this regard.

Approximately one third (32%) indicated that Healthmail had a positive effect on medication adherence, with nearly twice as many perceiving it had no effect (61.9%). Whilst 65.2% indicated that Healthmail had a positive/strongly positive effect on medication safety, nearly half indicated that Healthmail had a negative/strongly negative effect on patient understanding and overview of their medicines (46.1%). Related to this, 63.2% thought it had led to patients feeling less involved in the decision-making related to their care. Nearly one quarter said it had a negative effect on patient autonomy (24.7%), with equal proportions indicating it had no effect or a positive effect (37.6%). Overall, most respondents (87.5%) indicated that having electronic prescriptions with repeats that go only to one pharmacy is good for the pharmacy, whilst 59.3% stated that it was good for the patient.

### 3.11 Implications for future practice

#### 3.11.1 Future communication

Overall, 94.7% agreed that routine EPT was a positive step for pharmacy practice, 96.1% agreed that EPT should continue, and 89.1% disagreed that we should return to physical paper prescriptions only. Most pharmacists agreed (91%) that electronically transferred prescriptions should have a universal template.

Most pharmacists (81.1%) would be comfortable moving to an almost completely paperless electronic prescribing system and 63% would be comfortable conducting almost all interventions with the prescriber via electronic communication in future.

On receipt of a prescription via Healthmail, 67.6% agreed they would like to receive an expected time that the patient/representative will come to the pharmacy to collect a dispensed prescription. However, only 29.1% agreed that they would welcome using Healthmail to communicate with patients, whilst over half disagreed (51.7%).

#### 3.11.2 Future electronic records

At least three quarters of pharmacists indicated they would welcome the receipt of healthcare information other than prescriptions via Healthmail (78.4%), would be in favour of pharmacists having access to patients' electronic health records in future (75.9%), and would welcome an electronic health record integrating the prescription and pharmacy dispensing records (81%). Just over half (52.9%) agreed that they would prefer if pharmacists could retrieve prescriptions from an electronic repository with patients' permission (i.e. a ‘*pull*’ model) rather than prescriptions being sent electronically to a nominated pharmacy (i.e. a ‘*push*’ model, as in Healthmail), whereas 23.2% disagreed.

### 3.12 Qualitative results – suggestions to improve the EPT process and additional views on issues addressed in the survey

For the survey's final two questions with open comment sections, 303 pharmacists provided suggestions to improve the EPT process (61.3% of total respondents), whilst 191 pharmacists provided additional views on issues addressed in the survey (38.7% of total). Five major themes were generated from the data, as detailed in Table 2, with subthemes and quotations displayed under each theme to help explain the findings.

**Table 2:** Main themes and subthemes from the pharmacists' views on issues addressed in the survey and suggestions to improve the EPT process.

Theme 1: Stakeholder education and training	
Respondents emphasised the importance of education to improve stakeholders' understanding of and experience with using Healthmail. Proper guidance around the use of Healthmail for patients, prescribers, and pharmacists would significantly alleviate some of the issues that were experienced. There was a desire for more training on the use of Healthmail and how to use it better in practice. **1.1 Patient education** Pharmacists expressed frustration that patients lacked awareness of how the EPT process worked. Many patients assumed that their prescriptions would be sent immediately to the pharmacy, which was not always the case (e.g. due to delays in doctors sending them), and/or that the pharmacy staff would have the prescription ready when the patient arrived at the pharmacy. Some patients were even expecting prescriptions to be ready when they had only just been speaking to the doctor. It was noted that some patients were disappointed on arrival to pharmacies when prescriptions were not ready. Providing education to patients on EPT and realistic expectations with prescription processing were repeatedly emphasised. Some commented that prescribers or their colleagues should be partly responsible for managing patients' expectations regarding prescription times while conducting their consultations. It was also suggested that a public information campaign could be used to make patients more aware about Healthmail, possibly coordinated by Ireland's pharmacy regulator. One pharmacist noted that their pharmacy took the initiative to create a flyer to educate patients about Healthmail, albeit unsure of its effect. **1.2 Prescriber education** Pharmacists noted that some prescribers did not understand the legal requirements of prescriptions sent via Healthmail. It was also indicated that prescribers were making errors by sending prescriptions via Healthmail for medications that patients did not require. Pharmacists perceived that they were expected to educate prescribers on Healthmail use and were dissatisfied with this, and respondents suggested that prescriber training in Healthmail use was needed. It was indicated that some types of prescribers (e.g. potentially those who are older or less digitally literate) were less likely to understand how to use Healthmail to send prescriptions electronically (e.g. as they scanned paper prescriptions rather than sending them through prescribing software integrated with Healthmail). Similarly, these groups may not have undertaken training and required more education on EPT.	“*We need huge education and process improvement with Healthmail*”. **[#210]** “*The biggest problem I have had is the lack of understanding by the prescriber and the patient of the work and time that is required between the prescriber pressing the send button for a Healthmail prescription and the prescription being ready for collection at the pharmacy*.” **[#124]** “*We could get training webinars on the use of Healthmail*” **[#80]** “*We need better training from the Healthmail team to utilise this service better*” **[#428]** “*Patients need to be given a realistic expectation on the length of time before the prescription is sent and prepared in the pharmacy. No more patients arriving in with phone stuck to their ear, talking to the doctor, and expecting the prescription to be ready*.” **[#4]** “*Prescribers should give patients a realistic time frame of when prescriptions will be sent over. Often patients will be disappointed when a prescription has not yet been received by the pharmacy*”. **[#10]** “*Patients should be advised by the prescriber that there may be a delay in sending the script,* e.g. *waiting for doctor's authorisation. And also that the pharmacy will need time to process the script in rotation*.” **[#277]** “*For better communication to patient at surgery level to explain waiting times for the prescription to be processed*”. **[#373]** “*Patient education is also needed – the prescription isn't automatically gotten ready just because it was sent in*.” **[#220]** “*I think the public need more information about Healthmail. Some patients think that once the doctor sends the prescription it is ready for collection. Perhaps the PSI could co-ordinate a public information campaign in relation to this*.” **[#184]** “*Patient education (we have done our own little flyer but not sure how effective it has been)*” **[#79]** “*Wish all GPs understand the legal requirements for Healthmailed Rx*.” **[#377]** “*Prescribers need better training on using it. I have often received prescriptions with past and present medication included on the prescription*”. **[#134]** “*As always, pharmacists were expected to manage and roll out the process and educate prescribers on requirements for operation of same. No priority given to responsibilities of prescribers to learn to use Healthmail properly for electronic prescribing*” **[#5]** “*I think older surgeries have been less receptive to Healthmail and require more education around Healthmail use (*e.g. *they are more likely to just scan paper Rxs and send them as an attachment)*”. **[#471]** “… *there seemed to be a lack of training amongst prescribers especially those who are used to hand-writing all scripts and where there is a lack of confidence in using computers*” **[#149]**
**Theme 2: New challenges for pharmacy staff**	
**2.1 Managing patient expectations** As some patients have lacked understanding of Healthmail and the prescription journey, this had led to pharmacists dealing with difficult patient interactions and feeling obligated to educate patients on EPT and manage their expectations regarding when prescriptions will be dispensed and collection times. **2.2 Printing prescriptions** With the requirement to dispense from an original hard copy of a prescription that must be kept for filing, pharmacists noted that printing the physical prescriptions from the Healthmail inbox had increased the pharmacy costs due to the need for additional paper, ink, and printers. This had led to frustration amongst pharmacists as no remuneration was offered for this added cost. Many felt this was unfair, alluding to the fact that GPs have been provided with prescription paper for prescriptions that are reimbursed. Respondents noted that pharmacies should be remunerated for paper and ink, and that printers of a suitable standard should be supplied to all pharmacies. It was also noted that the additional printing is contradictory with ‘green pharmacy’ approaches aiming to minimise negative environmental impacts. **2.3 Prescribers' engagement with Healthmail and communication difficulties** Whilst Healthmail permits pharmacists to contact prescribers via a new mechanism regarding patients and their medication, respondents expressed resentment and frustration regarding some prescribers' use of Healthmail and not using it as a bi-directional communication system. Pharmacists stressed their desire for prescribers to have their inboxes actively monitored for queries so that they can be dealt with in a timely manner. Pharmacists have had to wait a long time for replies from GPs and this had disrupted pharmacy workflow. Pharmacists resorted to ringing the practices and speaking to the secretary staff, which was quite time consuming. Many respondents felt uptake of Healthmail for EPT had not been sufficiently widespread amongst prescribers. It was suggested that more hospital prescribers and dentists should utilise Healthmail for EPT. **2.4 Prescriptions transferred to the wrong pharmacy** Prescriptions were often sent to the wrong pharmacies, which led to issues when patients requested prescriptions and posing threats to patient confidentiality. Thereafter, pharmacists found difficulties when trying to get the prescription reissued to the correct pharmacy. Another resulting inconvenience was that some pharmacies were preparing the prescriptions that were unintentionally sent to them and then patients were not arriving to collect them. It was suggested that GPs and their colleagues should receive more training to minimise the number of times a prescription is sent to the wrong pharmacy. **2.5 Sender of prescription unclear and prescription access concerns** It was highlighted that pharmacists may not know definitively who the prescription sender is. Many pharmacists perceived that some Healthmail prescriptions were sent by administrative staff rather than prescribers, and expressed concern regarding access to patients' confidential records and potential medication errors that could be in patients' prescriptions – especially if administrative staff had not potentially consulted with a prescriber prior to sending. One pharmacist noted that any potential hacking of the Healthmail system could allow access to patient information and result in people losing confidence in the system's security.	*“…patients feel that once the Rx is ordered with the doctor and transmitted to the pharmacy, it will automatically be ready whenever they decide to collect it*” **[#185]** “*Patient expectations of how quickly a Healthmail can be processed is a recurring issue*” **[#456]** “*Patients' expectations need to be more realistic with regards to collection times*.” **[#134]** “*We need ink and paper and lots of it*” **[#428]** *“Cost of paper and ink for printing multiple copies for dispensing is a major negative impact of electronic prescriptions*” **[#218]** “*We need to find a way to negate the need to print paper copies as it is so unnecessary, and the PSI need to be told to accept this*” **[#250]** “*Stationery costs and paper claim requirements need to be addressed*” **[#25]** “*I really think that printing costs have ballooned for pharmacies but have decreased for GPs*” **[#236]** “*Pharmacists not been properly reimbursed for all the paper we are using. It's the underbelly of the green pharmacy*” **[#440]** “*Doctors should reply to our queries directed* via *Healthmail, it is not a one-way system and their disregard of our replies infuriates me*.” **[#37]** “*Doctors sometimes use Healthmail just to send prescriptions - without monitoring the inbox for incoming queries etc from the pharmacy*” **[#109]** “*GPs should check their Healthmail regularly for pharmacy queries especially now since they don't answer their phones as regularly*” **[#331]** “*Prescribers don't monitor for replies, and we end up having to phone the secretary which is very onerous and time consuming especially with surgery phone lines being so busy*” **[#418]** “*All pharmacies have been obligated to engage with Healthmail. GPs/hospitals have not. Please obligate them*” **[#327]** “*A lot of hospital consultants & hospital doctors, dentists are not aware of Healthmail at all. Healthmail need to proactively get them to register*.” **[#265]** “*Most common problem, until patients and doctors get used to system is getting Rxs for other pharmacies/ Drs sending Rx intended for us to different pharmacies*”. **[#470]** “*We have also noticed an increase in GDPR* [General Data Protection Regulation] *breaches with Healthmail scripts being sent to wrong pharmacies*” **[#11]** “*…a very large number of patients were not collecting their prescriptions which we realised after a few months was mainly private patients where the GP had sent to the wrong pharmacy*” **[#79]** “*We find GPs not willing to resend scripts that may have gone to the wrong pharmacy*” **[#76]** “*Better training for GP and receptionists so that scripts not sent to the wrong pharmacy*.” **[#488]** “*There is no way of knowing if the prescriber is signing off on regular prescriptions or it is a non-medically trained member of staff copying off patient files. It's possible a receptionist is logging in and could send prescriptions for anything without consulting the doctor at all*”. **[#110]** “*I worry doctor is not seeing Rx, only receptionist/secretary sends repeats* etc.” **[#278]** “*Who sends Rx? Dr or receptionist? Are surgeries just copying and pasting and pressing send?*”. **[#155]** “*I worry about secretaries having access to Healthmail accounts, I have experienced them forwarding prescriptions that have not been reviewed by a doctor first*”. **[#55]** “*Secretaries send the prescriptions a lot of the time*”. **[#288]** “*…if there was ever a hacking, the damage to confidentiality of patients and they would lose confidence in us and the system*” **[#59]**
**Theme 3: Implications for patients**	
**3.1 Patient-prescriber relationship** Pharmacists noted the importance of patients being reviewed by their doctors in person, and expressed concern that EPT had resulted in patients not attending their doctor in person as frequently and reduced patient-doctor communication. It was perceived that this may be leading to a lack of familiarity between patients and their doctors, and the prescription of unnecessary items. **3.2 Patient awareness of prescription content and autonomy** Due to direct transmission from prescriber to pharmacy, it was described how some patients were uncertain of what was on the prescription sent to the pharmacy or did not know at all. Some of this was perceived to be due to insufficient communication between patients and prescribers. It was suggested that this issue could be improved upon by incorporating a notification system that alerts the patient to what is on their prescription. It was felt that EPT is challenging patient autonomy, because access to their prescription is hindered. It was suggested that patients be provided with knowledge of what is on their prescription and greater ‘control’ so that they can feel more empowered in managing their health. It was also suggested that the prescription be accessible to patients so that they can note what item(s) they want before dispensing.	“*Patients need physical interaction with doctors*” **[#430]** “*Healthmail should not be a means of limiting or replacing patients' physical appointments with their GP*” **[#472]** “*Because doctors don't see and don't know their patients, Healthmail has led to massive increase in misprescribing (especially antibiotics) and over prescribing*” **[#56]** “*The biggest issue is around patients requesting their Rx from the GP and it's then sent by Healthmail but that patient doesn't actually need or expect these items to be dispensed - the rx should be sent to the patient to annotate what they want etc with when they want it to be dispensed before being sent to us…*” **[#27]** “*GPs and their patients often have communication breakdown. Patients have phone consult with doctor and expect prescriptions ready within minutes whereas for non urgent rxs it takes many practices 48 h to follow up. Patients should be educated that non urgent rxs will take time*” **[#132]** *“I think the patient should be notified of the content of their prescription - perhaps in a separate email to the patient (in the form of a notification as opposed to an Rx)*” **[#471]** “*Taking away a patient's right to have access to their prescription is wrong. Patients should be actively involved in their treatment. Electronic prescribing decreases this, at the moment anyway due phone consultations; very often they just receive call to say script sent but don't know what it's for*” **[#419]** “*Patients to have knowledge and control re their Rx*” **[#164]** “*Patients not aware what's on rx, lack of ownership over their own health*” **[#284]**
**Theme 4: Prescription information, format, and errors**	
**4.1 Prescription errors and their correction** Pharmacists expressed frustration with new types of errors that have come about with the use of Healthmail. Pharmacists' workload increased as prescribers were often including items on the prescription that were not required; this was attributed to prescribers unintentionally including previous items from a patient's medication record and/or not checking with patients about what items were required. As Healthmail had made it easier to copy old prescriptions, pharmacists expressed worry that prescribers were paying less attention to what they were prescribing for patients. Pharmacists noted the issue that prescriptions could only be amended after they were printed. As the prescription is stored in the Healthmail inbox, it was highlighted when it comes to repeat prescriptions that dispensing staff may re-print the email without realising it needs to be amended. Suggestions made by pharmacists included adding a mechanism to correct these errors electronically, which is not permitted with the current file formats, as this would allow pharmacists to amend the online prescription once checked with the prescriber. **4.2 Prescription file format** Pharmacists welcomed the introduction of the electronic prescription format as they are now more legible. However, when prescribers scanned physical prescriptions into the computer and sent them this way, this created issues with some prescriptions being harder to read. Varying file formats also resulted in differences between prescription layouts once printed. **4.3 Required and desired information and prescription standardisation** Pharmacists wanted additional patient details to be included in every prescription, such as their phone number and date of birth. Inclusion of these personal details would aid pharmacists when searching for a prescription and allow pharmacists to contact patients prior to collection to ensure they can prepare what the prescription needs. It was also noted that better search functionality was desired for Healthmail. Many of the legal prescription requirements were not being fulfilled, which was particularly evident for controlled drugs; this caused frustration amongst pharmacists. Suggestions to improve this included creating a template that could only be sent once all prescription requirements were met. Having a standardised email template would aid searching for prescriptions and streamline processes overall.	“*I have found more error prescriptions since Healthmail*” **[#97]** “*Prescribers should send only the medications needed at any given time requested by the patient instead of their full PMR* [Patient Medical Record] *for the previous 6 months (or longer!) which greatly increases the workload of the pharmacy when items have to removed from the bag and record when the patient comes to pick up and says ‘I don't need this, this and this’*” **[#226]** “*Worry that process is almost too automated in some surgeries, non-doctors just pressing send, over-reliance on the items the patient orders opposed to what should be on Rx* [prescription]” **[#155]** “*Huge issue with errors on repeat rxs*. *Cannot amend the Rx* [prescription] *in the email so if an error is made it may be dispensed incorrectly in subsequent months*” **[#194]** “*…facility to correct errors*” **[#140]** “*It's great that the prescriptions are legible*” **[#54]** “*Remove the option for prescribers to scan illegible typewritten or handwritten scripts as a Healthmail script*” **[#36]** *“Some GPs send as PDF, some send as inline text…formatting when printing therefore vary”***[#315]** “*Including patient contact details to see when they will be collecting and what they will be collecting*” **[#3]** “*If we had patient contact details we could text or call them when script is ready rather than constant phone calls asking when a script has arrived and when it going to be ready*” **[#227]** “*Much much better search facility, it's very poor*” **[#210]** “*Having a big issue with controlled drugs being written not in accordance with legislation and with repeats… Same issue repeatedly*” **[#418]** “*Controlled drugs should have a specific format which will not allow the prescriber to send the prescription until all relevant details are entered*” **[#468]** “*I think a universal template would streamline the Rx journey even further and improve compliance around Rx legalities*” **[#471]** “*Electronically transferred prescriptions should have a universal template and universal legal standards*” **[#235]** “*Standardised layout of emails to streamline search function*” **[#25]**
**Theme 5: Implementation of an optimised electronic prescription system**	
**5.1 The ‘push’ system has been an advancement, but ‘pull’ system preferred** Pharmacists indicated that Healthmail's EPT process has been a significant advancement for community pharmacy, but that it was “*overdue*” and should have been introduced years ago. Pharmacists felt that a ‘pull’ model, where a prescription could be retrieved from an online repository, would be preferred over the current ‘push’ model where prescribers send prescriptions directly to pharmacies. Pharmacists noted problems with the ‘push’ nature of Healthmail, such as reducing patient autonomy and prescriptions being sent to the wrong pharmacy (with prescriptions being duplicated and/or being prepared and not collected). It was seen as an advantage in having a ‘pull’ system as it would require a patient to request a prescription from a particular pharmacy, as well as minimising users' sending errors. **5.2 Integration with dispensing software** Pharmacists outlined that the need to dispense from physical prescriptions was something they would like eliminated. Many suggestions were made for the option of a system that was integrated with dispensing software that allowed the prescription to be downloaded and dispensed all on the same software without the need for a physical prescription. It was acknowledged that this integration would facilitate more ‘paperless’ approaches. **5.3 Electronic prescription endorsement and reimbursement** It was indicated that EPT could lead to the end of paper prescription requirements, and could better facilitate both the electronic endorsement and reimbursement of prescriptions. This would be welcomed by pharmacists as it would eliminate the need for printing of prescriptions and their subsequent filing, which would reduce the associated administrative burden (a process they perceived as ‘cumbersome’), as well as having a beneficial impact on the environment by reducing paper use. **5.4 Centralised patient records** Pharmacists indicated that they would welcome having a system that not only had electronic prescriptions integrated with dispensary software, but one that contained all patients' health information that healthcare professionals could access when information was required. If a system like this was used, it was indicated that pharmacists should receive permission from patients to access such a centralised health record. Having prescriptions electronically all in one central place would facilitate pharmacy staff in seeing what prescription items patients have received from elsewhere in the past.	“*Electronic prescribing is the way forward and should have been introduced years ago. In all my time working as a pharmacist, it is the single best advancement to be made without question*”. **[#119]** “*Has been the biggest step forward in dispensing since the computer-based records were introduced. Should have happened twenty years ago!*” **[#16]** “*Great advances but urgent ongoing need for pull model of rx distribution ASAP* [as soon as possible] *as overdue by decade or two*” **[#175]** “*Overall, electronic prescribing has been a huge advance in community pharmacy but the Healthmail push model badly needs to be modified*” **[#389]** “*Pull rather than push is the big one*” **[#143]** “*…we have experience of GP sending prescription to another pharmacy incorrectly… This is why I would support the pull system more than the push*” **[#418]** “*Should be ‘pull system’ where patient decides pharmacy but also to stop sending of emails to other shops and possible duplications*” **[#284]** “*That the patient gives the pharmacy the authority to pull the prescription rather than the patient relying on the doctor's secretary to send it correctly*” **[#84]** “*It needs to integrate with dispensary systems*”. **[#13]** “*Has to be integrated with dispensary computer and must be pull mode*.” **[#101]** “*Would like to see us going paperless. Retrieve the prescription directly to the patient's file and dispense from this*” **[#16]** “*Download Rx straight into dispensing software from Healthmail*” **[#57]** “*Integrating with dispensary software and abolishing requirement to print and endorse*.” **[#108]** “*Integrate Healthmail with electronic claims so you can do all* in silico” **[#127]** “*Being able to process and record the dispensing and claim for same without having to print any paper copy of the script*” **[#352]** “*Next stop should be electronic claim submission if we go paperless. Electronic stamping/ endorsing*” **[#209]** “*Hopefully no submission of paperwork to PCRS* [part of health service involved in reimbursing pharmacies for prescriptions dispensed] *– makes bundles large and cumbersome*”. **[#364]** “*I think patients should be given a unique patient number which would be used by all health care professionals, hospitals to enter all health information relating to that patient*” **[#468]** “*Centralised repository of prescriptions accessed by pharmacists on direction from patients*” **[#487]** “*A central spine with pharmacies and surgeries and hospitals having access to patients' information*” **[#448]** “*I think that the pull method of patients giving permission to a pharmacy to dispense a script or retrieve it is a good idea and amazed it's not been done a long time ago*”. **[#380]** “*Retrieve the Rx from a database and having access to previous dispensed medication to see the brand used and last time dispensed*” **[#438]**

# represents the pharmacist respondent number. EPT: electronic prescription transfer; GP: general practitioner; PCRS: Primary Care Reimbursement Service; Rx: prescription.

## 4 Discussion

This study is the first to evaluate community pharmacists' perspectives on the implementation of a national EPT system to pharmacies in Ireland. While the respondents had many suggestions to enhance EPT development, pharmacists seemed satisfied overall with using Healthmail for EPT – perceiving it as an easy-to-use, efficient, and convenient method of prescription transfer that integrated effectively into pharmacy dispensary workflows. Moreover, the majority of respondents indicated that Healthmail was the preferred method of prescription transfer and that it has enhanced communication overall between pharmacists and prescribers. Healthmail was particularly viewed as favourable with respect to patient safety, with most pharmacists perceiving improved prescription legibility, which has been shown previously.^30^ Given the established contribution of illegible prescriptions to dispensing errors and the challenges poor interprofessional communication poses to the correction of medication errors, Healthmail appeared to mitigate these issues, thereby contributing to improved patient care and satisfaction.31., 32., 33., 34., 35. Despite these benefits, several challenges related to the implementation of Healthmail for EPT were identified. Some pharmacists expressed concern that EPT may reduce patient involvement and limit patients' awareness of their prescribed medications, which has been a concern raised in the literature.^36^ Additionally, pharmacists believed that there had been insufficient information and training prior to the EPT rollout and a lack of clear guidance from professional regulatory bodies. When similar future systems are being implemented, stakeholders should receive sufficient education and training on new processes and how to optimise their integration.^37^

Pharmacists valued being able to review prescriptions before patients arrived, allowing additional time to identify prescribing errors early and improving workflow efficiency. However, the EPT introduction has brought with it increased patient expectations, with respondents indicating that patients expect prescriptions to be ready for collection upon their arrival. Additionally, pharmacists noted an increase in medications being dispensed but not collected following EPT implementation, possibly due to patient misunderstanding about how EPT works.^36^ Returning these dispensed medications brings about an increased workload through ‘rework’; an observational study from Ireland in 2021 found that approximately 5% of reworks in community pharmacies were due to prescriptions being prepared and not collected after they had been sent via Healthmail.^38^ To prevent issues like this, all stakeholders should be educated on new systems like this, as greater training in electronic prescribing improves efficiency.^39^ Furthermore, increased communication between pharmacies and patients using email or texting could help ensure patient expectations regarding wait times are managed, and encouraging medication adherence.40., 41. For any future initiatives like this both in Ireland and abroad, keeping affected parties informed where possible at different stages of the prescription transfer process is more ideal, coupled with education and training for all key stakeholders to manage expectations and enhance workflow efficiencies.

Pharmacists in this study perceived that Healthmail's EPT had a positive effect on medication safety. This may be because Healthmail has nearly eliminated the need for handwritten prescriptions, decreasing the occurrence of illegible prescriptions – a known contributor to dispensing errors.42., 43. Pharmacists also perceived that Healthmail's EPT has had a somewhat positive effect on patients' medication compliance. This may be because compliance is encouraged due to the direct transmission of prescriptions from prescribers to pharmacies, thus reducing some hassle for patients to collect prescriptions from the prescriber.44., 45., 46. Beyond the patient benefits, this study also illuminated some potentially negative impacts on patients. In particular, pharmacists perceived that patients' understanding of their prescribed medications and patients' involvement in their care have decreased. EPT has been shown to reduce face-to-face interactions with prescribers and thus patients may not have a thorough discussion on vital information regarding medication, such as indication, dosage instructions, and side effects.36., 47. For future research, it would be valuable to specifically investigate Healthmail's impact on patients' medication management.

Other than patient benefits, pharmacists also reported Healthmail's positive influence on pharmacist-prescriber relations. Pharmacists indicated that communication with prescribers has improved overall since the introduction of Healthmail, which was found to be particularly difficult during the COVID pandemic.48., 49., 50. Enhanced communication has been previously shown to improve the ease of medication queries through electronic means between healthcare professionals.37., 51. Furthermore, this enhanced pharmacist-physician communication has also been shown to improve medication information accuracy upon admission to hospital when compared to the traditional physical method of prescribing.^52^ However, these communication benefits were not universal. Pharmacists noted that for Healthmail to be more effective, physicians (or a delegate, like a GP's secretary) need to actively monitor their inboxes, a practice that was seemingly not consistently implemented. Going forward, clearer ETP procedures could improve standardisation and ensure pharmacists' queries are addressed promptly by the appropriate prescriber.^35^ Using email-based systems for querying prescriptions sent electronically should help cut down waiting times for replies, particularly for non-urgent issues.53., 54. The present study found that 63% of pharmacists would be comfortable conducting all intervention queries through Healthmail in future, so this warrants universities to ensure this type of communication is addressed as part of the teaching and assessment when training pharmacy students.

Healthmail's financial burden must also be considered. Pharmacists noted that the cost of paper, ink, and printers was substantial given that pharmacies were required to print and retain a physical copy of the prescription for reimbursement. However, this printing requirement was eliminated in January 2026, facilitating a fully paperless prescription process through Healthmail. While integrated electronic prescribing systems have shown to reduce costs significantly,^55^ there should also be a positive environmental impact with this ‘greener pharmacy’ approach.56., 57.

This study is strengthened by its good response rate relative to other similar pharmacist surveys in Ireland.49., 58., 59. The absolute number of responses was lower than a previous survey regarding the Falsified Medicines Directive (FMD), which received 618 responses but was also sent to pharmacists registered in other settings (even though they had to work in community pharmacy settings at least occasionally).^60^ The high number of responses in the FMD survey may also have been due to respondents having strong negative feelings regarding the FMD, in contrast to the more positive views shown in this survey regarding Healthmail. Furthermore, the present survey's response rate may have been hindered due to its timing in the midst of a winter season during the COVID-19 pandemic.

With 61.3% of respondents providing suggestions to improve the EPT process, these findings will be particularly useful to help inform jurisdictions where EPT is not yet in place, for those aiming to optimise the EPT processes, as well as settings planning on transitioning to electronic prescribing systems. Countries that have only recently adopted EPT systems, such as Moldova, Germany, and Luxembourg, may particularly benefit from these findings, as the study identified practical implementation challenges associated with EPT alongside targeted recommendations for improvement, which may support optimisation during the early phases of implementation.61., 62., 63., 64. Lower- and middle-income countries which have yet to implement any EPT systems should particularly benefit from this study highlighting pertinent implementation barriers, such as lack of education around rollout and unclear communication pathways, so as to streamline future system rollouts.5., 65., 66. It is vital that there are learnings taken from the experiences of other countries, where a variety of EPT approaches have been employed,^67^ as well as the present research. The suggestions provided in this study should also be useful for countries like Ireland aiming to implement electronic prescribing with a centralised healthcare record that is accessible to pharmacy staff, with prescriptions retrieved via a ‘pull’ system rather than the likes of Healthmail's ‘push’ approach.67., 68. Pharmacist suggestions which should be considered for future practice include integrating the healthcare record with the dispensing software (or vice versa), as well as facilitating electronic prescription endorsement and reimbursement.69., 70., 71., 72. Given that the present study was conducted during the COVID-19 pandemic, it would be prudent to gather key stakeholders' views prior to the introduction of any optimised systems.

Perhaps the most critical area for future research is the feasibility of implementing electronic prescribing alongside an electronic health record accessible to pharmacies. The HSE, Ireland's healthcare services provider, has recognised Healthmail as a transitional step toward a fully integrated electronic prescribing system, making it essential that challenges identified in this study such as unclear guidance and lack of formal training during Healthmail implementation are addressed in the rollout of electronic prescribing.^73^ Moreover, the findings of this study offer valuable lessons for other jurisdictions considering the introduction of EPT systems.

## 5 Conclusion

This study has provided a novel exploration of pharmacists' views regarding the national implementation of a system to facilitate EPT from prescribers to community pharmacies. While pharmacists were mostly positive about EPT introduction, they highlighted issues such as the lack of understanding by patients and prescribers regarding the process, managing patients' expectations, its financial impact on pharmacies, and reduced patient contact with their prescriber and prescription. This study has provided several useful solutions on how to deal with these challenges and outlined important suggested strategies which should be taken on board by software developers, healthcare professionals, patients, and policy makers – both for how EPT can be improved upon and for the development of electronic prescribing systems internationally in future.

## CRediT authorship contribution statement

**Kieran Dalton:** Writing – review & editing, Writing – original draft, Visualization, Supervision, Project administration, Methodology, Investigation, Formal analysis, Data curation, Conceptualization. **Ciara Kenny:** Writing – review & editing, Writing – original draft, Visualization, Methodology, Investigation, Formal analysis, Data curation, Conceptualization. **Maeve Culhane:** Writing – review & editing, Writing – original draft, Visualization, Formal analysis. **Robert Callaghan:** Writing – review & editing, Writing – original draft, Visualization, Formal analysis.

## Declaration of competing interest

None.
