# Supplementary material for: Community pharmacists’ views on the implementation of a national electronic prescription transfer system to pharmacies: A cross-sectional survey
Source: Explor Res Clin Soc Pharm. 2026 Jul 29;23:100826. doi: 10.1016/j.rcsop.2026.100826 (PMC13524745; doi:10.1016/j.rcsop.2026.100826)
Supplement: Supplementary file 1 [file mmc1.docx]

**Appendix A: Survey**

**Demographics**

1. What is your gender?
2. Please select your age range

- Age 25 or less
- Age 26-35
- Age 36-45
- Age 46-55
- Age 56-65
- Age 66+

3. How many years of post-registration experience do you have in community pharmacy?

- Less than 1 year
- 1-3 years
- 4-10 years
- 11-20 years
- 21-30 years
- 31+

4. Select the community pharmacy role(s) that currently apply to you. Tick all that apply

- Pharmacy Owner
- Superintendent Pharmacist
- Supervising Pharmacist
- Pharmacy Store Manager
- Support Pharmacist
- Locum Pharmacist
- Other(s)

5. Which pharmacy setting do you most frequently work in?

- An independent pharmacy
- A pharmacy which is part of a small chain (Less than 10 pharmacies)
- A pharmacy which is part of a large chain (10 or more pharmacies)

6. Please select the description that best applies to the location of the pharmacy where you most frequently work.

- Rural/village
- Town/suburb
- City
- Shopping centre (located in a town/city)
- Shopping centre (located on the periphery of a town/city)
- Other

7. What is the approximate distance from the pharmacy in which you most frequently work to the nearest GP clinic?

- Located in the same building
- Within 100m
- Within 500m
- Within 1km
- Within 2km
- Within 5km
- Within 10km
- Greater than 10km

8. How would you describe your confidence using technological devices (e.g. computers)?

- Extremely confident
- Very confident
- Moderately confident
- Slightly confident
- Not at all confident

**The Pharmacist’s Experience of using Healthmail**

9. At present, in the pharmacy in which you most frequently work:

Out of every 10 prescriptions that are dispensed, how many prescriptions have been sent via Healthmail?

1	2	3	4	5	6	7	8	9	10

10. At present, in the pharmacy in which you most frequently work:

Out of every 10 hospital prescriptions received, how many prescriptions have been sent via Healthmail?

1	2	3	4	5	6	7	8	9	10

11. Regarding when you and your team check the pharmacy Healthmail account in the pharmacy where you most frequently work:

Please rank the following options from 1 to 4, where 1 represents the option that best describes when the team check the Healthmail account and 4 represents the option that least applies.

- At set times during the day
- Periodically
- When the alert sounds
- When a patient requests a prescription and they inform us it has been sent to the pharmacy via Healthmail

12. Regarding the staff member who most commonly checks the Healthmail account in the pharmacy in which you most frequently work:

Please rank the following options from 1-7, where 1 represents the staff member who most commonly checks the Healthmail account and 7 represents the option that least applies. Any options that are not applicable should be ranked below the ‘Not applicable’ option.

- I do
- Another pharmacist
- Pharmaceutical Assistant
- Pharmacy Technician
- Pharmacy Intern
- Pharmacy assistant
- Not applicable

13. At present, in the pharmacy in which you most frequently work:

Out of every 10 prescriptions issued via Healthmail, how many prescriptions do pharmacy staff see BEFORE the patient (or patient representative) requests its dispensing?

1	2	3	4	5	6	7	8	9	10

14. When pharmacy staff see a Healthmail prescription BEFORE the patient or patient representative presents to the pharmacy, how does this impact the service you provide? Please indicate to what extent you agree with each of the following statements.

|  | Strongly agree | Agree | Neither agree nor disagree | Disagree | Strongly disagree |
| --- | --- | --- | --- | --- | --- |
| Improved patient safety |  |  |  |  |  |
| Improved patient satisfaction |  |  |  |  |  |
| If the item is not in stock, we can inform the patient of this before they present to the pharmacy |  |  |  |  |  |
| We feel more prepared to counsel the patient or patient representative |  |  |  |  |  |
| I have noticed an increase in prescription items being dispensed and not collected |  |  |  |  |  |

15. In relation to the impact Healthmail has had on your work processes in the community pharmacy setting, please select the most appropriate option in response to the following statements.

|  | Strongly agree | Agree | Neither agree nor disagree | Disagree | Strongly disagree |
| --- | --- | --- | --- | --- | --- |
| Healthmail is an efficient and convenient method of prescription transfer |  |  |  |  |  |
| I have found Healthmail easy to use overall |  |  |  |  |  |
| Healthmail has added to my workload |  |  |  |  |  |
| Healthmail has added to the workload of my colleagues |  |  |  |  |  |
| I feel the Healthmail system has been successfully integrated into the workflow of the dispensary |  |  |  |  |  |
| Healthmail has helped to streamline the dispensing process |  |  |  |  |  |
| I feel more at risk of making dispensing errors when dispensing prescriptions issued by Healthmail |  |  |  |  |  |
| The pharmacy I most frequently work in has a designated SOP for Healthmail/electronic prescription transfer. |  |  |  |  |  |
| Healthmail has helped in the implementation of infection control procedures in the pharmacy (due to COVID-19) |  |  |  |  |  |
| I prefer receiving prescriptions via Healthmail/electronic prescription transfer than other methods (excluding for infection control reasons) |  |  |  |  |  |

16. Regarding communication between pharmacists and prescribers, please select the most appropriate option in response to the following statements.

|  | Strongly agree | Agree | Neither agree nor disagree | Disagree | Strongly disagree |
| --- | --- | --- | --- | --- | --- |
| Healthmail has improved communication between pharmacists and prescribers |  |  |  |  |  |
| Prescriptions issued by Healthmail are easier to read than physical paper prescriptions |  |  |  |  |  |
| I have noticed less medication-related errors made by prescribers on prescriptions issued by Healthmail in comparison to physical prescriptions (handwritten or typed) |  |  |  |  |  |
| Prescriptions issued by Healthmail often lack one or more of the legal requirements for prescriptions in Ireland |  |  |  |  |  |
| Prescriptions for Controlled Drugs issued by Healthmail generally meet all legal requirements |  |  |  |  |  |
| I often have difficulty identifying the prescriber on prescriptions issued by Healthmail (e.g. missing or incorrect prescriber name) |  |  |  |  |  |
| Prescribers include extra information with prescriptions issued by Healthmail to improve patient care (e.g. information regarding dose changes) more commonly than with physical prescriptions |  |  |  |  |  |
| Other than prescriptions, I have received other confidential patient information via Healthmail that I did not request (e.g. laboratory parameters) |  |  |  |  |  |
| Healthmail has worked well in the context of electronic prescription transfer from ‘out of hours’ services (e.g. Caredoc /SouthDoc/D-Doc etc.) |  |  |  |  |  |

17. For this survey, an intervention is defined as "any communication between a pharmacist and a clinician, whether verbal or written, made with the intention of influencing prescribing".

Regarding the number of interventions you have made with the prescriber: in comparison to physical paper prescriptions, there has been:

- A large increase in the number of interventions with Healthmail
- A small increase in the number of interventions with Healthmail
- No change in the number of interventions with Healthmail
- A small decrease in the number of interventions with Healthmail
- A large decrease in the number of interventions with Healthmail

18. I have replied to a Healthmail to conduct an intervention with the prescriber.

Yes / No

-If the respondent selects ‘Yes’, they will be prompted to answer question 19 and then move on to question 21.

If they select ‘No’, they will be moved to question 20 and then on to question 21.

19. In relation to conducting interventions via Healthmail, please select the extent to which you agree or disagree with the following statements.

|  | Strongly agree | Agree | Neither agree nor disagree | Disagree | Strongly disagree |
| --- | --- | --- | --- | --- | --- |
| In general, the interventions I have conducted via Healthmail have had the same outcome as interventions conducted by traditional methods (e.g. telephone). |  |  |  |  |  |
| In general, a resolution is reached in a timely manner when using Healthmail to conduct interventions. |  |  |  |  |  |
| For some interventions I have made via Healthmail, I have not received any response from the prescriber and I have had to take further action (e.g. telephone call). |  |  |  |  |  |

20. What are your reasons for choosing not to conduct an intervention with the prescriber via Healthmail? Please tick all that apply.

- I feel that electronic communication is not an effective way to conduct interventions with prescribers
- Concerns regarding patient confidentiality
- I don’t think I would get a response in a timely manner
- I know that the prescribers I most commonly communicate with do not respond to pharmacist interventions via Healthmail
- I don’t think a satisfactory resolution could be reached using electronic communication
- Other (please specify)

21. In relation to conducting interventions with the prescriber, please indicate to what extent you agree or disagree with the following statements.

|  | Strongly agree | Agree | Neither agree nor disagree | Disagree | Strongly disagree |
| --- | --- | --- | --- | --- | --- |
| I would be comfortable conducting almost all interventions with prescribers via electronic communication methods in future. |  |  |  |  |  |
| Electronic communication with prescribers is an appropriate way to conduct minor interventions (e.g. missing legal requirement not related to a controlled drug). |  |  |  |  |  |
| Electronic communication with prescribers is an appropriate way to deal with clinical issues (e.g. drug-drug interaction). |  |  |  |  |  |
| Electronic communication is an appropriate way to contact prescribers when the matter is urgent. |  |  |  |  |  |

22. Regarding the security of the Healthmail system, please select the most appropriate option in response to the following statements.

|  | Strongly agree | Agree | Neither agree nor disagree | Disagree | Strongly disagree |
| --- | --- | --- | --- | --- | --- |
| Healthmail is a secure method of prescription transfer. |  |  |  |  |  |
| Healthmail has reduced the number of prescriptions that have ‘gone missing’ or ‘gotten lost’. |  |  |  |  |  |
| Prescriptions issued by Healthmail are less likely to be forged. |  |  |  |  |  |
| Healthmail increases the risk of people inappropriately accessing confidential patient information e.g. other staff members in the healthcare setting. |  |  |  |  |  |
| I worry that paperless prescriptions may facilitate other people inappropriately requesting and collecting medications not meant for them. |  |  |  |  |  |

23. In relation to record-keeping and reimbursement, please select the most appropriate option in response to the following statements.

|  | Strongly agree | Agree | Neither agree nor disagree | Disagree | Strongly disagree |
| --- | --- | --- | --- | --- | --- |
| Healthmail has made complying with record-keeping requirements more difficult. |  |  |  |  |  |
| Pharmacies should be remunerated for printing supplies associated with the use of Healthmail. |  |  |  |  |  |
| Prescriptions issued by Healthmail have streamlined procedures for obtaining reimbursement from the government (i.e. end-of-month claim). |  |  |  |  |  |

24. Regarding ongoing training and support on the use of Healthmail, please select the most appropriate option in response to the following statements.

|  | Strongly agree | Agree | Neither agree nor disagree | Disagree | Strongly disagree |
| --- | --- | --- | --- | --- | --- |
| I have experienced a lot of technical issues using Healthmail in the past month. |  |  |  |  |  |
| I received adequate information and training on the use of Healthmail (e.g. Healthmail website FAQs, HSE circulars, IIOP webinars etc.). |  |  |  |  |  |
| The joint HSE/PSI/Medical Council guidance for pharmacists and prescribers on the electronic transfer of prescriptions via Healthmail was easy to follow |  |  |  |  |  |

25. How would you rate the quality of ongoing support provided by the Healthmail team?

- Very helpful
- Helpful
- Not at all helpful
- Not applicable/not needed

26. Another pharmacy has emailed a prescription to your pharmacy via Healthmail at the request of the patient as it has been sent to them in error. How would you deal with this situation?

Please select the option you feel is most appropriate.

- I am happy to dispense the medication from the email sent to me from the other pharmacy.
- I am not happy to dispense the medication, so I ask the patient to contact the prescriber to send me the prescription via Healthmail before I dispense the medication.
- I am not happy to dispense the medication, so I contact the prescriber myself to ask them to send me the prescription via Healthmail before I dispense the medication.
- I am not happy to dispense the medication, so I ask the patient to go to another pharmacy.

**Impact of Healthmail on Patient**

27. Please rate the degree of impact/effect that you believe that electronic prescription transfer (i.e. Healthmail) has had on the following factors:

|  | Strongly positive effect | Positive effect | No effect | Negative effect | Strongly negative effect |
| --- | --- | --- | --- | --- | --- |
| Medication safety |  |  |  |  |  |
| Medication adherence |  |  |  |  |  |
| Patient autonomy |  |  |  |  |  |
| Patient understanding and overview of their medications  (e.g. knowing content of prescription / number of repeats left / when to return to prescriber for check-up) |  |  |  |  |  |
| Communication between pharmacy staff and patients |  |  |  |  |  |

28. Having electronic prescriptions with repeats that go only to one pharmacy is:

|  | Strongly agree | Agree | Neither agree nor disagree | Disagree | Strongly disagree |
| --- | --- | --- | --- | --- | --- |
| Good for the patient |  |  |  |  |  |
| Good for the pharmacy |  |  |  |  |  |

29. In relation to the impact you think Healthmail has had on patients, please indicate to what extent you agree or disagree with the following statement.

|  | Strongly agree | Agree | Neither agree nor disagree | Disagree | Strongly disagree |
| --- | --- | --- | --- | --- | --- |
| I feel that Healthmail has led to improved patient satisfaction. |  |  |  |  |  |
| Healthmail has facilitated a decrease in patient waiting times in the pharmacy. |  |  |  |  |  |
| Healthmail has resulted in patients expecting their prescriptions to be dispensed more quickly |  |  |  |  |  |
| I feel that patients do not have enough information about Healthmail |  |  |  |  |  |
| I think patients have adapted well to the use of Healthmail |  |  |  |  |  |
| I think Healthmail has led to patients feeling less involved in the decision-making related to their care |  |  |  |  |  |
| I think patients will become less aware of the legal requirement for a prescription as a result of electronic prescription transfer |  |  |  |  |  |

30. Compared to the time spent on patient consultations where a physical paper prescription is handed in, there is:

- A large increase in time spent on patient consultations for Healthmail prescriptions
- A small increase in time spent on patient consultations for Healthmail prescriptions
- No change in time spent on patient consultations for Healthmail prescriptions
- A small decrease in time spent on patient consultations for Healthmail prescriptions
- A large decrease in time spent on patient consultations for Healthmail prescriptions

**Implications for future practice**

31. In relation to your views on the impact of Healthmail on future practice, please select the most appropriate option, in response to the following statements

|  | Strongly agree | Agree | Neither agree nor disagree | Disagree | Strongly disagree |
| --- | --- | --- | --- | --- | --- |
| Overall, the routine electronic transfer of prescriptions is a positive step for pharmacy practice |  |  |  |  |  |
| Electronically transferred prescriptions should have a universal template where possible |  |  |  |  |  |
| On receipt of a prescription via Healthmail, I would like to simultaneously receive an approximate expected time that the patient/representative is going to come to the pharmacy to collect the dispensed medication |  |  |  |  |  |
| I think we should return to physical paper prescriptions only |  |  |  |  |  |
| I believe electronic prescription transfer should continue |  |  |  |  |  |
| I welcome the receipt of healthcare information other than prescriptions via Healthmail (e.g. diagnoses, patient test results) |  |  |  |  |  |
| I would welcome using Healthmail to communicate with patients |  |  |  |  |  |
| I would be in favour of pharmacists having access to patients’ electronic health records in future |  |  |  |  |  |
| I would welcome an electronic health record integrating the prescription and the pharmacy dispensing records |  |  |  |  |  |
| I would be comfortable moving to an almost completely paperless electronic prescribing system |  |  |  |  |  |
| Rather than prescriptions being sent electronically to a nominated pharmacy (i.e. a 'push' model), I would prefer if pharmacists could retrieve prescriptions from an electronic repository with patients' permission (i.e. a 'pull' model). |  |  |  |  |  |

32. How could the process of electronic prescription transfer be improved? Please provide up to 3 suggestions.
33. Please include any other thoughts/comments regarding issues addressed in this survey.
